# Genetic hitchhiking, mitonuclear coadaptation, and the origins of mt DNA barcode gaps

**DOI:** 10.1002/ece3.6640

**Published:** 2020-08-03

**Authors:** Geoffrey E. Hill

**Affiliations:** ^1^ Department of Biological Science Auburn University Auburn AL USA

**Keywords:** neutral theory, purifying selection, recombination, selective sweeps, speciation

## Abstract

DNA barcoding based on mitochondrial (mt) nucleotide sequences is an enigma. Neutral models of mt evolution predict DNA barcoding cannot work for recently diverged taxa, and yet, mt DNA barcoding accurately delimits species for many bilaterian animals. Meanwhile, mt DNA barcoding often fails for plants and fungi. I propose that because mt gene products must cofunction with nuclear gene products, the evolution of mt genomes is best understood with full consideration of the two environments that impose selective pressure on mt genes: the external environment and the internal genomic environment. Moreover, it is critical to fully consider the potential for adaptive evolution of not just protein products of mt genes but also of mt transfer RNAs and mt ribosomal RNAs. The tight linkage of genes on mt genomes that do not engage in recombination could facilitate selective sweeps whenever there is positive selection on any element in the mt genome, leading to the purging of mt genetic diversity within a population and to the rapid fixation of novel mt DNA sequences. Accordingly, the most important factor determining whether or not mt DNA sequences diagnose species boundaries may be the extent to which the mt chromosomes engage in recombination.

## Graphical abstract

Neutral models of mitochondrial evolution predict mitochondrial DNA barcoding cannot work for recently diverged taxa, but mt DNA barcoding accurately delimits species across a wide spectrum of bilaterian animals. I propose that the tight linkage of genes on mt genomes that do not engage in recombination could facilitate selective sweeps whenever there is positive selection on any element in the mt genome, leading to the purging of mt genetic diversity within a population and to the rapid fixation of novel mt DNA sequences. If selective sweeps play a key role in generating mt DNA barcode gaps, then the most important factor determining whether or not mt DNA sequences diagnose species boundaries within a taxon would be the extent to which the mt chromosomes of individuals within that taxon engage in recombination.

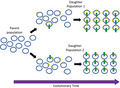

## 1 INTRODUCTION

Bilaterian animals carry mitochondrial (mt) genotypes that typically show little variation among individuals within a species but with substantial variation between species (Bucklin, Steinke, & Blanco‐Bercial, 2011; Dasmahapatra & Mallet, 2006; Lane, 2009b; Zahiri et al., 2014). There is some debate regarding how reliably mt DNA genotypes diagnose species, particularly closely related species (Dupuis, Roe, & Sperling, 2012; Ratnasingham & Hebert, 2013), but for birds (Tavares & Baker, 2008), mammals (Clare, Lim, Engstrom, Eger, & Hebert, 2007), turtles (Naro‐Maciel et al., 2010), bony fish (Ward & Holmes, 2007), amphibians (Vences, Thomas, Bonett, & Vieites, 2005), spiders (Coddington et al., 2016), butterflies (Janzen et al., 2009), ants (Smith, Fisher, & Hebert, 2005), parasitoid wasps (Smith et al., 2008), mayflies, stoneflies, and caddisflies (Morinière et al., 2017), among other bilaterian taxa, mt genotypes change abruptly at the great majority of species boundaries. This pattern of differentiation in mt genes among species has led to the use of mt nucleotide sequences as a diagnostic tool in species identification, the so‐called “DNA barcode” (Hebert, Cywinska, Ball, & deWaard, 2003). The substantial divergence in mt DNA sequence observed between most sister pairs of animal taxa is termed the “barcode gap” (Hebert, Cywinska, et al., 2003).

Three mechanisms have been proposed to explain this pattern of diversity of mt genotypes: (a) Variations in mt DNA nucleotide sequences are neutral and are fixed via drift in isolated populations (Hickerson, Meyer, & Moritz, 2006; Lynch, Koskella, & Schaack, 2006; Moritz, Dowling, & Brown, 1987; Smith, 2016; Zink & Barrowclough, 2008), (b) there have been repeated episodes of extreme population bottlenecks involving the majority of bilaterian taxa (Stoeckle & Thaler, 2014), or (c) directional selection on mitochondrial genotypes leads to rapid divergence when gene flow between populations is disrupted (Chou & Leu, 2010; Gershoni, Templeton, & Mishmar, 2009; Hill, 2016; James, Piganeau, & Eyre‐Walker, 2016). In this essay, I focus on the necessity of coadaptation with the nuclear (N) genome throughout the evolution of the mt genome as a foundation for explaining the population structure of mt genomes. I propose that, far from being an unexpected or inexplicable pattern, the tight congruence between mt genotype and species boundaries may be an inevitable consequence of the need for mt and N gene products to cofunction to enable aerobic respiration, especially when the mt chromosome does not engage in recombination. In reviewing previous efforts to explain DNA barcode gaps, I consider the nearly exclusive focus on amino acid substitutions and the protein‐coding genes of the mt genome as potential targets of selection, which has discounted the potential key role played by selection on genes coding for transfer RNA (tRNA) and ribosomal RNA (rRNA) as well as selection on origin of replication regions of mt DNA (Adrion, White, & Montooth, 2016; Barreto & Burton, 2013; Barreto et al., 2018; Ellison & Burton, 2008a; Ruiz‐Pesini & Wallace, 2006). I also consider a potentially pivotal role played by recombination of mitochondrial chromosomes in the generation of mt DNA barcode gaps. I propose that a better understanding of the evolutionary mechanism that generates the genetic structure of mt DNA across eukaryotes is critical not only with regard to assessing the value of DNA barcodes as a tool in taxonomy (Baker, Tavares, & Elbourne, 2009; Rubinoff, Cameron, & Will, 2006) but also for a better understanding of the process of speciation (Hill, 2016; Sunnucks, Morales, Lamb, Pavlova, & Greening, 2017; Tobler, Barts, & Greenway, 2019).

## 2 NEUTRAL MODELS

It was long assumed that the great majority of the evolution of mt genomes was neutral and hence that genetic structure of mt DNA within and among populations was necessarily the product of drift (Avise, 2004; Ballard & Kreitman, 1995; Lynch et al., 2006). The assumption of neutrality in changes to mt genotypes emerges from the recognition that all protein‐coding genes in the animal mt genome code for subunits of the electron transport system and therefore that the protein products of the mt genome are among the most system‐critical proteins in the entire animal genome (Bar‐Yaacov, Blumberg, & Mishmar, 2012; Lane, 2011). Functional changes to such mission‐critical genes were proposed to be so rare as to be realistically ignored, leaving the assumption that observed evolutionary changes in the mt genome will be neutral (Saccone et al., 2000). The rapid coalescence of mt genotype compared to N genotype in populations of eukaryotes was proposed to arise as a simple consequence of the small effective population size of the mt genome in relation to the N genome—a result of the mt genome being haploid and maternally transmitted (Hickerson et al., 2006; Palumbi, Cipriano, & Hare, 2001; Zink & Barrowclough, 2008).

Arguments for using mt DNA as a neutral marker of evolution rested on the assumption that essentially all selection on mitochondrial genotypes would be in the form of purifying selection to maintain the current forms of mt‐encoded proteins with no functional change in gene products and with no functional variation between groups (Rand, Dorfsman, & Kann, 1994; Stewart, Freyer, Elson, & Larsson, 2008). Synonymous changes to the nucleotide sequence, which are defined as changes that do not affect the amino acid sequence of a protein, were predicted to evolve via genetic drift and thus to accumulate across evolutionary time at a rate proportional to population size (Lynch et al., 2006; Stoeckle & Thaler, 2014; Wilson et al., 1985). However, fundamental predictions of the neutral hypothesis for mitochondrial evolution have not been supported. Neutral theory predicts that genetic variation within a population should be proportional to the size of that population. Contrary to this prediction, there is no consistent relationship between population size and variation in mt DNA sequence (Bazin, Glémin, & Galtier, 2006; Nabholz, Glemin, & Galtier, 2009; Stoeckle & Thaler, 2014). Moreover, the fixation of distinct mt genotypes between populations of at least some vertebrates (for which the rates of mutation of mt DNA are fairly well characterized) seems to occur much faster than predicted by neutral theory (Ballard & Whitlock, 2004; Hickerson et al., 2006). And finally, in contradiction to neutral theory, isolation by distance is unreliable for mt DNA (Teske et al., 2018). All things considered, neutral theory does not seem like the place to begin an investigation of the evolution of mt DNA and the origins of the mt DNA barcode gap (Kern & Hahn, 2018).

## 3 DEMOGRAPHIC BOTTLENECKS

In a recent essay, Stoeckle and Thaler (2014) posed the question: “A universal selection‐driven mtDNA clock implies all organisms are evolving at about the same rate.... What could cause similar rates of change for diverse organisms in diverse environments?” Stoeckle and Thaler (2014) proposed that the external environment of organisms could cause periodic extreme reductions in the population sizes of essentially all organisms, perhaps particularly at the point of divergence of incipient sister species. Severe demographic bottlenecks in population size would purge populations of genetic diversity in mt genotype and fix differences between species, potentially creating the pattern of mt DNA barcode gaps observed in bilaterian animals (Stoeckle & Thaler, 2014). No explanation is given for why such bottlenecks would reduce variation in mt genotypes but not N genotypes. Moreover, this hypothesis requires that, at regular intervals that average a few hundred thousand years, essentially every species is subjected to an extreme population bottleneck (Stoeckle & Thaler, 2014). These authors speculated that complex “food web, predator‐prey, and parasite‐host interactions” might sum to a common selective pressure on animal mitochondrial genomes “with long‐term planetary climate cycles as the ultimate driver of evolution” (Stoeckle & Thaler, 2014). I know of no evidence for such periodic synchronized collapse of all populations of all bilaterian organisms to create the pattern of mt DNA barcode gaps observed in bilaterian animals.

## 4 CHANGE IN mt DNA GENOTYPE VIA SELECTION

An alternative hypothesis to both neutral drift and demographic bottlenecks for the generation of mt DNA barcode gaps between species is directional selection on mt genotypes. Natural selection has the potential to shape the mt genome in response to two distinct environments: the external environment (both biotic and abiotic) and the internal genomic environment created by the N genome (Barreto et al., 2018; Hill, 2019a; Rand, Haney, & Fry, 2004; Sloan et al., 2018; Zhu, Ingelmo, & Rand, 2014). There is now evidence that the mt genome of at least some animal lineages is subject to periods of directional selection as adaptive responses to the external environment (Ballard & Pichaud, 2014; Dowling, Friberg, & Lindell, 2008; Kazancioǧlu & Arnqvist, 2014). In particular, thermal and chemical environments, oxygen pressure, diet, salinity, and UV exposure can all exert natural selection on the mt genome and lead to adaptive changes in protein‐coding genes (Ballard & Pichaud, 2014; Hill, 2019a). The adaptive evolution of mt genomes in response to external environments is now a major research topic in evolutionary biology (Hill et al., 2019; Sunnucks et al., 2017), and such changes to the nucleotide sequence of mitochondria in response to directional selection pose a serious challenge to core arguments for why mt DNA sequences will often fail as a tool for diagnosing species (Hickerson et al., 2006). Adaptive divergence of mt genotype in response to external environment is a key reason why mt DNA is predicted to rapidly diverge between allopatric populations (Gershoni et al., 2009; Tobler et al., 2019).

Perhaps even more important, and certainly more pervasive, than changes to mt DNA gene sequence in response to external environment is the potential for perpetual evolutionary change in the mt DNA in response to changes in the internal genomic environment (Barreto et al., 2018; Burton & Barreto, 2012; Chou & Leu, 2010; Hill, 2020; Sloan et al., 2018). The coadaptation of gene complexes is a foundational concept in evolutionary biology (Dobzhansky, 1937; Wright, 1942). In a discussion of the evolution of mt genomes, however, it is essential to grasp that there are unique features to the co‐evolution and coadaptation between mt gene products and the products of a small list of N genes that code for products that function in intimate interaction with mt gene products (N‐mt genes; Hill, 2019a; Shtolz & Mishmar, 2019). First, the system that depends on coadaptation of mt and N‐mt genes—the electron transport system—is the most critical biochemical system in the bodies of eukaryotes that depend on energy from aerobic respiration (Lane, 2014; Wallace, 2010). Second, because of the complexity of the ETS in controlling the flow of electrons and pumping of protons, very small changes to interacting components can have huge fitness effects (Hill, 2019a; Hill et al., 2019; Lane, 2011; Sloan et al., 2018). Third, mitonuclear coadaptation involves two genomes that can potentially undergo independent evolution (Gershoni et al., 2014; Rand et al., 2004; Wolff, Ladoukakis, Enríquez, & Dowling, 2014). Fourth and finally, the mt genome of animals does not generally engage in recombination (Barr, Neiman, & Taylor, 2005) and so mitochondrial genes form one linkage group such that selection on one mt gene can affect the frequencies of other mt genes (Meiklejohn, Montooth, & Rand, 2007; Oliveira, Raychoudhury, Lavrov, & Werren, 2008). Functional divergence in mt DNA will be particularly effective in creating Dobzhansky–Muller incompatibilities in hybrid offspring and hence in establishing barriers to gene flow because the mt DNA must maintain tight coadaptation with the N genome (Burton & Barreto, 2012; Hill, 2017).

If changes in mt genotype between species were entirely neutral, then matching the N genes of one species with the mt genes of a closely related species—either through hybridization or in cell culture by directly manipulating genomes—should result in no change in mitochondrial function in the resulting cells or organisms. Indeed, this logical extension of the neutral theory of mitochondrial evolution led to a failed research program to propagate endangered species by pairing mitochondria of donor species to the N genome of the species to be saved (Lanza, Dresser, & Damiani, 2000). Observations from cybrid and hybrid studies, however, clearly established that, once sets of mt and N‐mt genes diverge in nucleotide sequences to the extent seen in sister species, incompatibilities in non‐coadapted gene sets cause a reduction in mitochondrial function when they are forced to work together (reviewed in Hill, 2019a). Mitonuclear incompatibilities in cybrid cells and hybrid organisms are strong evidence that the evolution of mt genotypes is not neutral with respect to the genomic environment (Barrientos, Kenyon, & Moraes, 1998; Ellison & Burton, 2008b; Garvin, Bielawski, & Gharrett, 2011; Latorre‐Pellicer et al., 2016; Lee et al., 2008).

The evolution of uniquely coadapted mt and N‐mt genotypes is a critical concept because it potentially explains both how the mt genotypes of sister species rapidly diverge and why there is so little introgression of divergent mt genotypes between species within most clades of bilaterian animals (Burton & Barreto, 2012; Hill, 2016). The evolution of a clean mt DNA barcode gap requires that the propagation of population‐specific mitochondrial genotypes is constrained to remain within‐species boundaries across generations (Hebert, Ratnasingham, & Waard, 2003). Even a small amount of introgressive flow of mitochondrial genotypes, which would be inevitable under neutral models of mitochondrial evolution if species lived in sympatry, would add unacceptable ambiguity into barcoding efforts (Papadopoulou et al., 2008). In the rare cases in which mitochondria do introgress across species boundaries, the introgression tends to be rampant, with complete replacement of one mitochondrial genotype by another (Hill, 2019b). All of these patterns are consistent with a process whereby coevolution of mt and N‐mt genotypes leads to loss of fitness (at the level of the individual organism) when mt genotypes are paired to N‐mt genes to which they are not coadapted. The barcode gap is more than an arbitrary marker of species boundaries—it is the functional boundary that reinforces the uniqueness of a species’ mitonuclear genotype (Burton & Barreto, 2012; Chou & Leu, 2010; Hill, 2016, 2017; Lane, 2009a).

## 5 THE GENE CONTENT OF A BARCODE GAP

The pattern of little variation within a species but substantial variation between species is the reason that DNA barcoding is proposed as a useful tool for taxonomists (Meyer & Paulay, 2005). But what, specifically, are the fixed differences in nucleotide sequences that create barcode gaps? In vertebrates, including birds (Kerr, 2011), mammals (Tobe, Kitchener, & Linacre, 2010), and fish (Ward & Holmes, 2007), variation in amino acid sequence is rare in the barcoding region of the cytochrome c oxidase subunit one (COX1) gene. Thus, the barcode gap that is commonly observed using the conventional COX1 barcode gene (Kerr et al., 2007; Tavares & Baker, 2008) is comprised almost entirely of synonymous nucleotide changes, and there is evidence for strong purifying selection on the nonsynonymous nucleotide positions within the COX1 barcode gene (Kerr, 2011; Popadin, Nikolaev, Junier, Baranova, & Antonarakis, 2013; Stewart et al., 2008). In contradiction to the prediction that adaptive evolution of the COX1 gene might underlie the evolution of DNA barcode gaps (Hill, 2016), there is too little variation in the amino acid sequence of the product of the COX1 gene between sister taxa for this prediction to be correct (Kerr, 2011). The paradox of the COX1 barcode gene is that, despite departure from expectations of neutral theory, there seems to be little opportunity for adaptive divergence creating the differences among species in the nucleotide sequence of the COX1 barcode gene (Kwong, Srivathsan, Vaidya, & Meier, 2012). Certainly, there are a handful of very well‐documented cases of COX1 adaptively diverging between sister taxa in response to changes in the oxygen pressure (Luo, Yang, & Gao, 2013; Scott et al., 2011; Tomasco & Lessa, 2014) or hydrogen sulfide exposure (Greenway et al., 2020; Pfenninger et al., 2014) in the external environment. Such adaptive divergences in COX1 genotype, however, cannot account for the barcode gap that has been documented between thousands of sister taxa.

A paucity of nonsynonymous changes in the barcode region of the COX1 gene is not difficult to explain. COX1 is the least changeable gene in the entire mitochondrial genome (da Fonseca, Johnson, O’Brien, Ramos, & Antunes, 2008; Kerr, 2011). The conserved nature of COX1 is a major reason that it was chosen as the barcode gene: Primer sets developed for model species tend to work for nonmodel species (Hebert, Cywinska, et al., 2003). COX1 is one of thirteen protein subunits of Complex IV of the ETS, which is the rate‐controlling enzyme in the OXPHOS system (Arnold, 2012; Pacelli et al., 2011), and COX1 holds the key catalytic position of that crucial enzyme (Wang & Pollock, 2007). Thus, Complex IV is a particularly critical enzyme in animal systems that depend on aerobic respiration, and the barcode gene, COX1, is the most critical subunit of this most critical enzyme (Pierron et al., 2012). I propose that the resolution of this paradox of species‐specific variation in the COX1 barcode sequence without functional changes in the COX1 gene lies in the tight linkage of genes on the mitochondrial chromosome and genetic hitchhiking of neutral substitution in the COX1 barcoding region with adaptive changes in other regions of the mt genome (Meiklejohn et al., 2007).

## 6 SYNONYMOUS/NONSYNONYMOUS VERSUS FUNCTIONAL/NONFUNCTIONAL

For most pairs of sister species that recently evolved from a common ancestor and now have a DNA barcode gap, there is no difference in the amino acid sequence of the portion of the COX1 gene serving as the barcode gene (Kwong et al., 2012). However, this does not necessarily mean that there are no functional changes to other protein‐coding genes that include seven subunits of Complex I, one subunit of Complex III, two (additional) subunits of Complex IV, and two subunits of Complex V. Indeed, the seven mitochondrially encoded protein subunits of Complex I are much more frequently implicated in adaptive divergences between sister taxa than Complex IV subunits (da Fonseca et al., 2008; Garvin, Bielawski, Sazanov, & Gharrett, 2014). At least some sister taxa also carry fixed differences in amino acid sequence for subunits of Complexes III and V (reviewed in Hill, 2019a). A hypothesis that is worthy of testing is that the pattern of little variation within species but substantial differences between species in mt DNA sequence arises entirely as a consequence of strong selection on adaptive amino acid substitutions in mt‐encoded proteins (da Fonseca et al., 2008). Given available data, however, I do not think that an adaptive protein evolution hypothesis will be the primary solution to the paradox of the mt DNA barcode gap, because purifying selection is, indisputably, the dominant force in the evolution of all mt protein‐coding genes (Kerr, 2011; Stewart et al., 2008).

I propose that the key to explaining the evolution of the mt DNA barcode gap lies in giving full consideration to the fact that all of the genes encoded by the animal mitochondrial genome will evolve via natural selection primarily in response to the internal genomic environment (Hill et al., 2019; Sloan et al., 2018; Sunnucks et al., 2017). Most of the genes in the mitochondrial genome code from products other than proteins; in most bilaterian animals, 24 out of 37 mitochondrial genes (65%) code for tRNA or rRNA (Burton & Barreto, 2012; Rand et al., 2004). Every one of these genes maintains coadaptation with the N‐encoded genes through coevolution; in other words, there is a prediction of perpetual directional adaptive evolution of all of the products of the mt genome in response to the internal genomic environment (Hill, 2019a; Kivisild et al., 2006; Wei et al., 2019; Zaidi & Makova, 2019). The selective driver of this process of adaptive evolution of mt genes is compensatory coevolution, whereby N genes evolve so as to compensate for deleterious mt genotypes and vice versa (Barreto et al., 2018; Dowling et al., 2008; Hill, 2020; Osada & Akashi, 2012; Rand et al., 2004). Even the noncoding region of the animal mitochondrial DNA, which serves as the origin of replication site for transcription and replication, coevolves with N genes (Ellison & Burton, 2008a, 2010; Gaspari, Falkenberg, Larsson, & Gustafsson, 2004). The expression of mt and N genes that code for cofunctioning units must also be coregulated, another important level of mitonuclear coadaptation (Barshad, Blumberg, Cohen, & Mishmar, 2018; Calvo et al., 2020).

There is a large and rapidly growing literature showing that single‐nucleotide substitutions in each of the non‐protein‐coding genes of the animal mitochondrion have important fitness consequences (reviewed in Hill, 2019). Some of these fitness effects play out in relation to the external environment of the organism (Hoekstra, Siddiq, & Montooth, 2013), but the source of the hypothesized perpetual evolutionary change of all of the products of the mt genome would be selection to maintain coadaptation with products of the N genome to enable cellular respiration (Barreto et al., 2018; Hill, 2020; Meiklejohn et al., 2013). Because it is dependent on random mutations in both the N and mt genomes, coevolution of cofunctioning mt and N genes to maintain mitochondrial function will be idiosyncratic, unpredictable, and not repeatable (Blount, Lenski, & Losos, 2018). Directional selection on both the mt and N genomes to maintain mitonuclear coadaptation will create the sort of divergence in mt genotypes between species that give rise to a DNA barcode gap (Burton & Barreto, 2012; Hill, 2016). The key missing element is: How would divergence in a tRNA, rRNA, or the control region affect the barcode region of the COX1 gene or other synonymous substitutions in protein‐coding genes?

## 7 GENETIC HITCHHIKING

**Figure 1:**
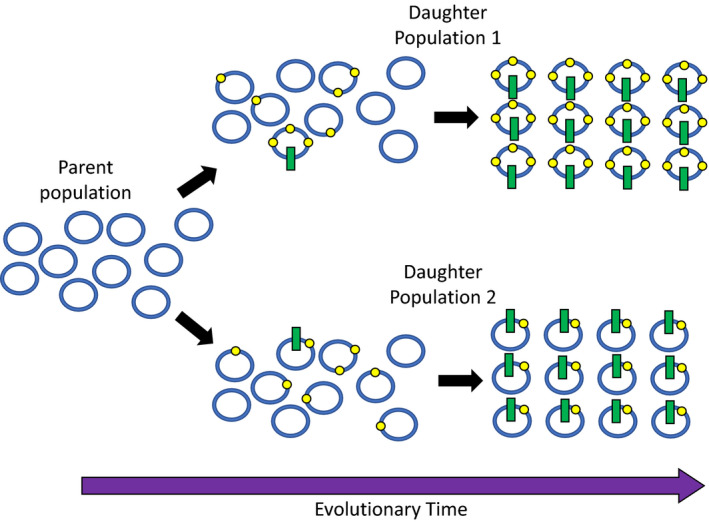
An illustration of rapid evolution of divergent mitochondrial genotypes between allopatric daughter populations via genetic hitchhiking. Blue rings represent the mitochondrial genome of an individual in a population. Yellow dots represent functionally neutral changes to the nucleotide sequence. Green bars represent functional changes that bestow a benefit and that are subject to positive selection. Selection on the beneficial alleles leads to the fixation of those beneficial alleles along with any neutral traits that happen to be linked to them while at the same time purging the population of genetic variants

The animal mitochondrial genome is a single, effectively non‐recombining chromosome, and the genes on this chromosome form one linkage group (Gray, 1999). Under such circumstances, genetic hitchhiking is inevitable (Maynard Smith & Haigh, 1974). Genetic hitchhiking results when strong positive selection on one genetic element causes an increase in the frequency of not only the element under selection but also all of the genetic elements to which it is linked (Gillespie, 2000; Meiklejohn et al., 2007; Figure 1). The implications of genetic hitchhiking for the creation of a mt DNA barcode gap are inescapable (Costa & Carvalho, 2010). If a favorable mutation occurs in any part of the mitochondrial genome—if for instance there is a nucleotide substitution in a mt‐tRNA that improves the speed and accuracy of translation of mRNA (Adrion et al., 2016)—then positive selection for that mutation would cause an increase in the frequency of *the entire mitochondrial genotype that held that mutation*. If the mt chromosome that carried that favorable allele happened to also carry a unique, neutral mutation in the barcoding region of the COX1 gene, then that COX1 mutation would rise in frequency along with the mt‐tRNA gene. Selection for the favorable allele could lead to rapid fixation of the new genotype, purging all diversity in mitochondrial genotypes within that population (Figure 1). This process of genetic hitchhiking would essentially pull the mitochondrial genotype through a series of bottlenecks that would simultaneously purge standing variation within a population and fix differences in mt genotype between populations, creating the pattern of barcode gaps that typify the genomic structure of animals (Barton, 2000; Maynard Smith & Haigh, 1974; Meiklejohn et al., 2007). Because the mt and N genomes are inherited independently and N genes engage in recombination each generation, N genes could escape the bottleneck events affecting gene frequency in the mt genome.

The power of this explanation is that the proposed process would be ubiquitous among animals. Across most bilaterian animals, the genes that contribute to the function of the electron transport system are rigidly conserved—the same N genes cofunction with the same mt genes in a fruit fly and a chimpanzee (Boore, 1999; Gissi, Iannelli, & Pesole, 2008). A common set of interacting genes that are subject to the same functional constraints is exactly the circumstance that would give rise to a universal, selection‐driven mt biological clock that runs faster than predicted by neutral theory (Hickerson et al., 2006). Adaptations to the external environment would only add noise to the dominant mode of evolution driven by mitonuclear coevolution.

Selective sweeps arising from the rapid fixation of mt variants under positive selection are a process already under discussion regarding the pattern of variation in mitochondrial genotypes within and among populations (Kerr, 2011; Meiklejohn et al., 2007). By adding a need to consider both the protein‐coding and noncoding genes of the mt genome to the list of genes likely to be subject to at least periodic positive selection, a much greater opportunity for frequent selection sweeps is recognized. The majority of gene products of the mt genome is tRNAs, and the rate of mutation and evolutionary change of tRNA is much greater than the rate of amino acid substitutions in protein‐coding genes (Thornlow et al., 2018). Moreover, changes to mitochondrial tRNAs can have large effects on function and fitness. Numerous maternally inherited mitochondrial diseases are caused by nucleotide substitution on genes coding for mt tRNAs (Suzuki, Nagao, & Suzuki, 2011) and effects in nonhuman animals have also been documented (Meiklejohn et al., 2013). Given that function of mt tRNAs is dependent on the genotype of N‐encoded aminoacyl tRNA synthetase and N‐encoded post‐transcriptional processing proteins, we would predict positive selection for better performing variants as well as negative selection for dysfunctional variants (Adrion et al., 2016; Pett & Lavrov, 2015). The same arguments for the importance of functional evolution of mitochondrial tRNAs also apply to mitochondrial‐encoded rRNA (Scheel & Hausdorf, 2014). Mitochondrial rRNA evolves at a rate that is an order of magnitude faster than the N‐encoded ribosomal proteins (Barreto & Burton, 2013), and these changes have functional consequences: As with tRNA, human inherited diseases are linked to nucleotide changes in mt rRNA (Scheper, van der Knaap, & Proud, 2007). Changes to the nucleotide sequence of the control region also can have functional consequences in terms of human disease (Chinnery et al., 2002), and functional divergence of the control region among sister taxa of animals can play a role in postzygotic isolation of populations (Ellison & Burton, 2010). Positive selection on any of these non‐protein‐coding genes should lead to selective sweeps that would fix neutral changes across the mitochondrial genome, including in DNA barcode regions, and this process would be perpetual and inevitable because of the necessity of coadaptation of the mitochondrial and N genomes.

Eyre‐Walker (2006) pointed out that there is an interesting interaction between population size, genetic diversity, and genetic hitchhiking. As the size of a population increases, the amount of genetic diversity contained within that population, both in the mt and N genomes, will increase. This increased within‐population diversity of mt genomes would work against the effectiveness of mt DNA barcodes for large populations. However, larger populations offer greater potential for the appearance of adaptive variants of mt genes and hence a greater opportunity for genetic hitchhiking and selective sweep. He suggested that these two opposing forces might tend to negate each other, leaving genetic diversity of mt (but not N) genotypes largely independent of population size.

## 8 SELECTION SWEEPS OF THE W CHROMOSOME OR mt DNA?

The W chromosomes of birds have very low rates of variation, suggesting that the genes on this chromosome have been subjected to selective sweeps (Berlin & Ellegren, 2004; Ellegren, 2013; Smeds et al., 2015). This low rate of variation in the genes on the avian W chromosome led Berlin, Tomaras, and Charlesworth (2007) to hypothesize that selective sweeps on genes in the W chromosome would also result in selective sweeps on the mt genome via genetic hitchhiking, due to strict maternal linkage (perfect cotransmission) of mt DNA and the W chromosome. Following this logic, Berlin et al. (2007) proposed that evidence of selective sweeps of the mt genome would be evidence for positive selection on W genes. However, it could also work the other way: Selective sweeps of the mt genome could result in genetic hitchhiking and loss of variation in the W chromosome (Lane, 2008; Marais, 2007). The fact that the W chromosome of all birds investigated shows signs of loss of genetic diversity via selective sweeps is thus, potentially, further support for the idea that the mt DNA barcode pattern is a consequence of selective sweeps. Birds are not the only taxa with ZW sex determination and cotransmission of mt and W chromosomes, but to date there are much more sequencing data available for the W chromosome of birds than for any other ZW taxa. A broader survey of genetic diversity of W‐linked genes might make it possible to distinguish whether selection on the W‐linked genes or mt‐linked genes is responsible for observed patterns of low genetic diversity.

## 9 COMPENSATION‐DRAFT FEEDBACK

The co‐evolution of mt and N genes has been proposed to lead to rapid serial fixation of alleles if a positive feedback loop arises as a consequence of changes and counterchanges between coevolving mt and N‐mt genes. This idea is called the compensation‐draft feedback hypothesis (Oliveira et al., 2008). Compensatory coevolution describes a situation whereby cofunctioning sets of mt and N‐mt genes are each under strong selection to improve aspects of performance that arise from the products of the other genomes (Hill, 2020). For instance, it was experimentally demonstrated in a laboratory population of nematodes that the mt genome rapidly evolved a novel genotype to compensate for an OXPHOS dysfunction created by a N‐mt allele (Christy et al., 2017). This example involves the interaction of protein‐coding genes in an experimental laboratory setting, but for the reasons stated above, in most natural populations, the interacting mt and N gene products may be involved in transcription, translation, and replication of mt genes. The rapid fixation of mt genomes that carried this single adaptive nucleotide change might also have led to fixation of slightly deleterious alleles that happened to be associated with that allele. By this process of compensation‐draft feedback, selective sweeps fix one problem while creating future problems that can be fixed through further selective sweeps when solutions happen to evolve. Such a series of selective sweeps would perpetually suppress within‐population variation in mt genotypes while rapidly generating unique mt nucleotide sequences among populations thereby giving rise to mt DNA barcode gaps.

## 10 AN EXPLANATION FOR WHY MITOCHONDRIAL BARCODING FAILS

DNA barcoding using sequences from mt‐encoded proteins or rRNA works very well for bilaterian animals, but it is much less effective in delimiting species boundaries of some other eukaryotic taxa, particularly plants (Chase et al., 2005; Kress, Wurdack, Zimmer, Weigt, & Janzen, 2005) and fungi (Xu, 2016) but also Porifera (sponges) and Anthozoa (corals and sea anemones; Huang, Meier, Todd, & Chou, 2008). Recombination of mt genomes, which is rare or nonexistent in bilaterian animals, slows down or stops selective sweeps because beneficial alleles can be fixed in a mt genotype independent of the frequencies of other genes on the mt chromosome (Charlesworth, Morgan, & Charlesworth, 1993; Rokas, Ladoukakis, & Zouros, 2003; White, Wolff, Pierson, & Gemmell, 2008). The hypothesis for the evolution of barcode gaps that I outline in this paper, therefore, provides testable hypotheses for why mt DNA barcoding might fail for some taxa. If the efficacy of barcoding is dependent on selective sweeps, which in turn is dependent on lack of recombination of mt genomes, then it follows that taxa with recombination of mt genes will have a poor mt DNA barcode signal. Intriguingly, the mt genomes of Porifera and Anthozoa, for which mt DNA barcoding also works poorly, include introns, have very low mutation rates, and likely engage in recombination (Brockman & McFadden, 2012; Gissi et al., 2008; Huang et al., 2008). Recombination of mitochondrial DNA has been documented in some plants and fungi (Barr et al., 2005), but the scope of recombination across these eukaryotic groups remains poorly known. For plants, the extent of recombination and the potential for selection sweeps are likely irrelevant to a failure of an effective mt DNA barcode—the rates of nucleotide substitution in plants (with some exceptions) are far lower than in other eukaryotic taxa, leaving little opportunity for the evolution of species‐specific mt genotypes (Cowan, Chase, Kress, & Savolainen, 2006). A broad‐scale comparison of the efficacy of mt DNA barcoding in relation to rates of recombination and nucleotide substitution of mt DNA could be very illuminating.

Rampant introgression of mt genomes, wherein the mitochondrial genotype of one species replaces the mt genotype of another species with little change to N genotypes, will also erase a barcode signal (Hill, 2019b; Toews & Brelsford, 2012). Such mt introgression is hypothesized to occur when (a) the fitness gain from a better adapted heterospecific mitochondrion compensates for fitness losses from mitonuclear incompatibilities, (b) escape from mutational erosion and loss of mt function compensate for loss of mitonuclear incompatibilities, or (c) a maternally transmitted parasite like *Wolbachia* infects a new host species and, because it is cotransmitted with mitochondria, causes the spread of the mt genotype of the original host species in the new host species (Hill, 2019b; Sloan, Havird, & Sharbrough, 2017). The effects of endosymbionts may be particularly problematic for the persistence of a mt DNA barcode gap because endosymbionts can drag mitochondria across a species boundary and could be an explanation for why phenotypically distinct populations of animals like blowflies (Diptera: Calliphoridae) which have high rates of infection by endosymbionts often share a mitochondrial genotype (Whitworth, Dawson, Magalon, & Baudry, 2007). Loss of a uniquely coadapted mitonuclear genotype could be viewed as loss of species identity such that a lack of a DNA barcode gap in cases of rampant mt introgression is correctly failing to diagnose a collapsed species (Vonlanthen et al., 2012). Such an argument carries a risk of circularity, but the congruence between mt DNA barcode gaps and both conventional species designations (Hebert, Ratnasingham, et al., 2003; Tavares & Baker, 2008) and distinct ornamentation used during mate choice for species recognition (Hill, 2018) establishes a clear link between transitions in mitochondrial genotype and real boundaries among populations. The cases of rampant introgression of mt genomes then become rare exceptions that can be explained within the context of the mitonuclear compatibility species concept (Hill, 2019b).

## 11 DIAGNOSING SPECIES WITH MITOCHONDRIAL GENES

There is a widely held view that the use of mt DNA to diagnose species is a certain‐to‐be‐flawed approximation that evolutionary biologists must endure until advances in sequencing technology allow us to do the job correctly with N genes. Because the mitochondrial genome is a single linkage group (at least for bilaterian animals), it is proposed that sequences from multiple N genes will reveal species boundaries with fundamentally better accuracy than will mt genes (Pazhenkova & Lukhtanov, 2019). For instance, Chase et al. (2005) wrote that we will advance from mt gene sequences to “more sophisticated barcoding tools, which would be multiple, low‐copy nuclear markers with sufficient genetic variability and PCR‐reliability” to “identify the ‘genetic gaps’ that are useful in assessing species limits.” Along the same lines, Edwards et al. (2005) commented that “in our view, maternally inherited mtDNA can never capture enough of a species’ history to delimit species on its own” and that “mtDNA should not have priority over N genes in avian species delimitation.” Furthermore, it is sometimes stated that mt genomes introgress across species boundaries more readily than N alleles (Bonnet, Leblois, Rousset, & Crochet, 2017). The success of DNA barcoding across the majority bilaterian animals is conspicuous evidence that introgression of mt genomes across species boundaries is a rare rather than a common event. As evolutionary biologists compare N genes and mt genes between closely related species of bilaterian animals, the typical pattern that emerges is that the boundaries revealed by N genes are fuzzy while the boundaries between mitochondrial genotypes are discrete (Barrowclough & Zink, 2009; Hill, 2019a; Petit & Excoffier, 2009; Toews et al., 2016). This pattern, of course, is why mt genes are used as DNA barcode genes. If mitonuclear interactions underlie the process of speciation, species limits are best defined by coadapted sets of cofunctioning mt and N‐mt genes, and a close proxy to this true species diagnosis is simply mt genotype (Hill, 2017).

## 12 SUMMARY

In many taxa of bilaterian animals, there is little diversity in mitochondrial genotype within a species but substantial variation between species. This pattern is the basis for mt DNA barcoding as a means for identifying species. Despite the failure of neutral theory to explain this pattern of mt DNA sequence variation, most of the variation in the nucleotide sequence of barcode genes is neutral with respect to function. In other words, changes to the nucleotide sequence of mt DNA are evolving in a non‐neutral manner despite the fact that they have no functional consequences. A solution to this paradox is that directional selection on any gene in the mitochondrial genome, including genes that code for rRNA and tRNA, can lead to selective sweeps that eliminate genetic diversity and fix neutral or slightly deleterious alleles in other parts of the mt genome. It is proposed that genetic hitchhiking by neutral elements in the DNA barcoding region explains how the DNA barcode gap evolves. This hypothesis proposes that mt DNA barcodes will only be effective when there is little or no recombination of mt genes, potentially explaining why mt DNA barcoding is ineffective for some groups of eukaryotes.

## CONFLICT OF INTEREST

The author declares no competing interests.

## AUTHOR CONTRIBUTION

**Geoffrey E. Hill:** Conceptualization (equal); Writing‐original draft (equal).

## Data Availability

No original data were used in writing this theoretical paper.
